# Decursinol-mediated antinociception and anti-allodynia in acute and neuropathic pain models in male mice: Tolerance and receptor profiling

**DOI:** 10.3389/fphar.2022.968976

**Published:** 2022-09-29

**Authors:** LaTaijah C. Crawford, Sangyub Kim, Deepkamal Karelia, Diana E. Sepulveda, Daniel J. Morgan, Junxuan Lü, Angela N. Henderson-Redmond

**Affiliations:** ^1^ Biomedical Sciences Graduate Program, Pennsylvania State University College of Medicine, Hershey, PA, United States; ^2^ Department of Biomedical Sciences, Marshall University, Huntington, WV, United States; ^3^ Department of Pharmacology, Pennsylvania State University College of Medicine, Hershey, PA, United States

**Keywords:** decursinol, neuropathic pain, antinociception, tolerance, mice

## Abstract

Korean scientists have shown that oral administration of *Angelica gigas* Nakai (AGN) root alcoholic extract and the metabolite of its pyranocoumarins, decursinol, have antinociceptive properties across various thermal and acute inflammatory pain models. The objectives of this study were 1) to assess whether tolerance develops to the antinociceptive effects of once-daily intraperitoneally administered decursinol (50 mg/kg) in acute thermal pain models, 2) to establish its anti-allodynic efficacy and potential tolerance development in a model of chemotherapy-evoked neuropathic pain (CENP) and 3) to probe the involvement of select receptors in mediating the pain-relieving effects with antagonists. The results show that decursinol induced antinociception in both the hot plate and tail-flick assays and reversed mechanical allodynia in mice with cisplatin-evoked neuropathic pain. Tolerance was detected to the antinociceptive effects of decursinol in the hot plate and tail-flick assays and to the anti-allodynic effects of decursinol in neuropathic mice. Pretreatment with either the 5-HT_2_ antagonist methysergide, the 5-HT_2A_ antagonist volinanserin, or the 5-HT_2C_ antagonist SB-242084 failed to attenuate decursinol-induced antinociception in the tail-flick assay. While pretreatment with the cannabinoid inverse agonists rimonabant and SR144528 failed to modify decursinol-induced anti-allodynia, pretreatment with the opioid antagonist naloxone partially attenuated the anti-allodynic effects of decursinol. In conclusion, our data support decursinol as an active phytochemical of AGN having both antinociceptive and anti-allodynic properties. Future work warrants a more critical investigation of potential receptor mechanisms as they are likely more complicated than initially reported.

## Introduction

Effective management of chronic pain, including chemotherapy-evoked neuropathic pain (CENP), represents a major unmet medical need as current pharmacologic modalities/therapies have limited efficacy and undesirable side effects. CENP is an adverse side effect of platinum- and taxane-derived anticancer drugs that is often dose-limiting in patients. Opioid pain medications can be effective in some patients for treating chronic pain conditions such as CENP. However, the use of prescription opioids for the treatment of chronic pain, including CENP, is associated with serious negative side effects including constipation, tolerance, dependence, use disorder, and overdose death (for a review, see Benyamin et al., 2008).

In 2020, 68,630 deaths from opioid overdose were reported by the Centers for Disease Control (CDC; Source: CDC Wonder Database) compared to 47,602 in 2017 and 46,802 deaths in 2018. Drug overdose data from 2020, as well as emerging data from 2021, suggest that the COVID-19 pandemic has strongly aggravated drug abuse and overdose (∼40% increase) in recent years. For many individuals with opioid use disorder, their initial exposure to an opioid was via prescription from their physician for treatment of pain. An unfortunate consequence of the reduction in opioid prescriptions that was implemented to address the current opioid crisis, is that many pain patients receive inadequate treatment. Therefore, alternative non-opioid pain treatments that are safer and non-addictive are urgently (desperately) needed.

**FIGURE 1:**
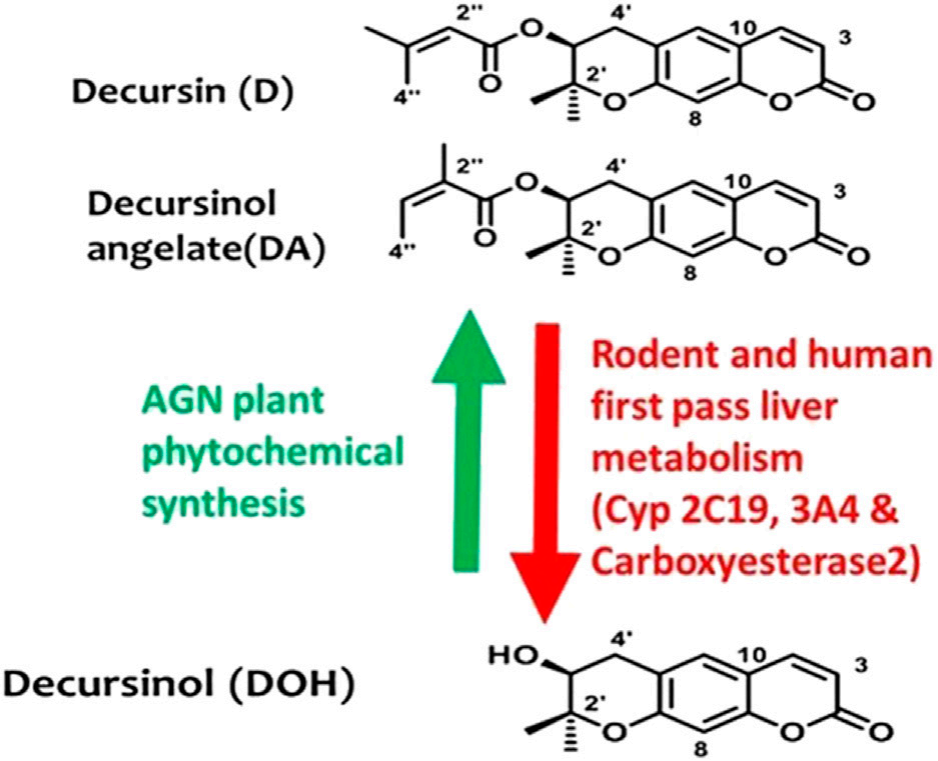
Chemical structures of morphine and the non-opioid alternative, decursinol. Chemical structures of AGN root signature pyranocoumarins in alcoholic extracts and their relationship in AGN plant phytochemical syntheses (Green arrow) vs. in mammalian pharmacology drug metabolism (Red arrow).

In traditional oriental medicine, *Angelica gigas Nakai* (AGN), known more commonly as the Korean angelica, has been used to treat many chronic disorders due to its anticancer (Ahn and Woong-Seop SimKim, 1995; Lü et al., 2015), anti-platelet (Yong et al., 2003), anti-inflammatory (Shehzad et al., 2016, 2018) antinociceptive (Choi et al., 2003b; Seo et al., 2009), and neuroprotective (Kang et al., 2001; Lee et al., 2003; Yan et al., 2004; So et al., 2005; Jiang et al., 2007; Seo et al., 2009) properties. The alcoholic extracts of AGN dried root contain the signature pyranocoumarins decursin (D) and its isomer decursinol angelate (DA; Figure 1) which is about 50–60% as abundant as D. Decursinol (DOH; Figure 1) is the precursor for the synthesis of D and DA and is detected at lower abundance than D and DA, if at all. In rodent models, orally administered (gavage) D and DA are rapidly and extensively converted to DOH through first-pass hepatic metabolism (Lü et al., 2015; Zhang et al., 2015) with a similar metabolism confirmed in humans (Lü et al., 2015; Zhang et al., 2015).

AGN herbal supplements have been tested in Korean patients for chronic pain as part of a small clinical trial (e.g., https://www.lifeextension.com/magazine/2007/7/report_korean_angelica). However, these results have not yet been published in a peer-reviewed journal. In the United States, AGN herbal supplements (such as “Decursinol-50” and “CognI.Q″) are currently marketed for minor pain and improved memory, respectively.

Previous studies by Korean scientists have demonstrated that oral administration of an alcoholic extract of the AGN root exerted dose-dependent antinociceptive effects in models of acute thermal pain and formalin-induced inflammatory pain (Choi et al., 2003a; 2003b). These researchers demonstrated that decursinol recapitulated the antinociceptive effects in these pain assays (Choi et al., 2003b) with an ED_50_ ∼ 50 mg/kg and a peak response 30 min after oral gavage. More recent work demonstrated that intrathecal injection of D (50 mg/kg) reversed mechanical allodynia associated with CENP caused by paclitaxel (Son et al., 2021). Earlier experiments using receptor antagonists suggest that the antinociceptive effects of decursinol might be mediated in part via noradrenergic, serotonergic, adenosine (A2), histamine H1, and histamine H2 receptors (Choi et al., 2003b). Interestingly, the effect of decursinol on thermal pain was not altered by the opioid receptor antagonist, naloxone, suggesting that these effects might not be mediated by opioid receptor signaling pathways.

In terms of rigor and reproducibility, neither the effects of AGN extract components on pain, nor investigations on the mechanism of action have been reported outside of Korea. Therefore, in the current study we sought to replicate the analgesic effects of decursinol in assays for both acute thermal (tail-flick and hot plate) and neuropathic (CENP) pain. Likewise, previous work demonstrated that methysergide, a non-selective serotonin (5-HT) receptor antagonist, could block the antinociceptive effects of decursinol (Choi et al., 2003b). As such, we used two selective 5-HT_2A_ and 5-HT_2C_ receptor antagonists to probe the specific 5-HT receptor subtype(s) responsible for these effects on pain and through which decursinol might be exerting its effects. As tolerance can negatively affect the clinical utility of novel analgesics, our ultimate goal was to determine if tolerance develops to the analgesic effects of decursinol in either acute and/or CENP pain models following prolonged administration to better assess its long-term therapeutic potential.

## Materials and methods

### Experimental animals

This study was performed using approximately 219 experimentally naïve eight- to ten-week-old male C57BL6/J mice. With preliminary work in our lab failing to find any sex differences in decursinol-induced antinociception, we exclusively used male mice that were either obtained from Jackson Laboratory or were the direct bred in house. Mice were group housed (3–5/cage) and maintained on a standard 12:12 h light/dark cycle (lights on at 06:00, lights off at 18:00) with *ad libitum* access to water and a standard chow diet. To ensure accurate dosing, mice were weighed prior to each drug injection. All animal procedures were carried out with the guidance of NIH guidelines for the care and use of laboratory animals and with approval from Pennsylvania State University’s (A3141-01) and Marshall University’s (A3578-01) Institutional Animal Care and Use Committees (IACUC).

### Drugs

Decursinol (442127) was purchased from Aktin Chemicals, Inc. (Chengdu, P.R. China) and the purity was verified to be higher than 98% by HPLC, ^1^H-NMR, and ^13^C-NMR. Decursinol was diluted into a 125 mg/ml stock solution using dimethyl sulfoxide (DMSO; 679; Fisher Scientific, Pittsburgh, PA). Methysergide maleate, a non-selective 5-HT_2_ receptor antagonist, was obtained from BOC Sciences (5281073; Shirley, NY) while the 5-HT_2A_ receptor antagonist volinanserin (5311271), the 5-HT_2C_ antagonist SB-242084 (3644637), the nonselective opioid antagonist naloxone hydrochloride (5464092), the CB_1_ inverse agonist rimonabant hydrochloride (104849), and the CB_2_ inverse agonist SR144528 (3081355) were all obtained from Cayman Chemical (Ann Arbor, MI). Methysergide was dissolved in DMSO, naloxone in saline, and all other drugs were dissolved in ethanol. Injection solutions were made from stock solution fresh each day of injection into either a saline (0.9% sodium chloride), 5% Cremophor El, 5% ethanol, and 4% DMSO solution (18:1:1 + 4% DMSO; decursinol and methysergide), saline (naloxone), or a saline and 5% Cremophor El (18:1; rimonabant and SR144528) solution. All drugs were administered by intraperitoneal injections (IP) in an injection volume of 10 ml/kg body weight.

### Hot plate testing

Hot plate nociception was assessed prior to tail-flick testing as previously described by our group using a Columbus Instruments hot plate apparatus (Columbus, OH) that was set to 55°C (Henderson-Redmond et al., 2016; Marcus et al., 2017; Nealon et al., 2019). A maximum cut-off time of 30 s was used to avoid tissue damage. Nociception was measured as the latency (seconds) to withdrawal (licking of paws or jumping) from placement on the hot plate. Measurements were made prior to (pre) and after (post) administration of drug. The percent maximal possible effect (% MPE) was calculated for both the hot plate and tail-flick nociceptive tests. The formula used to calculate the %MPE = [(post-drug latency–pre-drug-latency)/ (cutoff time-pre-drug latency)] x 100.

### Tail-flick testing

Tail-flick nociception was measured using a Columbus Instruments TF-1 analgesia meter (Columbus, OH) as previously described by our group (Morgan et al., 2014; Marcus et al., 2015; Nealon et al., 2019; Henderson-Redmond et al., 2020). Prior to testing, mice were acclimated to placement in a Broome-style restraint and placed on the apparatus. The tail-flick was calibrated to an intensity of 5 to elicit a baseline tail withdrawal latency of approximately 3–4 s in untreated wild-type mice. Nociception was measured as the latency (seconds) to elicit a withdrawal response (tail-flick) from the heat stimulus. To avoid tail tissue damage, a cut off time of 10 s was used. As with the hot plate test, results were reported in %MPE.

### Measurement of hypothermic effects

The hypothermic effects [% change in body temperature (%ΔBT)] of vehicle or drug were assessed using a mouse rectal thermometer probe (Physitemp, Clifton, NJ) to measure body temperature both before and after drug administration (Henderson-Redmond et al., 2016, 2021; Marcus et al., 2017; Nealon et al., 2018). Hypothermia was calculated using the formula: %∆BT = [(pre-drug temperature)- (post-drug temperature)]/ [(pre-drug temperature)] x 100.

### Cisplatin-induced neuropathy and von Frey testing

CENP was induced in male mice following four once-weekly IP injections with cisplatin (5 mg/kg; Nealon et al., 2019; Henderson-Redmond et al., 2020, 2021). Cisplatin (Tocris, Minneapolis, MN) was made fresh for each IP injection by diluting in saline (0.9% sodium chloride). Immediately after cisplatin administration, mice were given 1 ml of 4% sodium bicarbonate (Fisher Scientific, Pittsburgh, PA) in saline via subcutaneous (SC) injection to prevent nephrotoxicity (Guindon et al., 2014). Mechanical allodynia was assessed using an electro-von Frey anesthesiometer equipped with a semi-flexible polypropylene super tip (IITC Life Science Inc., Woodland Hills, CA). The electro-von Frey anesthesiometer was calibrated prior to testing using a 7-g weight. Mice were acclimated to the study room for 1 hour prior to testing, followed by a 20-min acclimation period in acrylic testing chambers (2.5″ x 4″ x 3.5”) placed on top of a wire mesh testing table. Mechanical allodynia was measured in triplicate using the right hind paw with a testing interval of approximately 5 min between the three testing trials and was calculated as an average paw withdrawal threshold (grams of force). Baseline von Frey measurements were taken prior to mice receiving any cisplatin. The development of CENP was confirmed by assessing mechanical allodynia with the von Frey test each week on the day prior to the next cisplatin injection. To measure tolerance, mice with CENP were first given an IP injection (day 0) of vehicle containing 5% ethanol, 5% Cremophor, and 4% DMSO in saline. After day 0, mice were administered either vehicle or decursinol (50 mg/kg) once-daily 30 min prior to von Frey testing. Rectal temperatures were measured before and after (∼40–50 min) vehicle or drug administration. Mechanical allodynia and hypothermia were measured each day for 15 consecutive days.

### Decursinol dose-response curves

#### Acute nociception dose-response

Male mice (N = 12) were administered increasing cumulative doses of decursinol [VEH (0), 10, 30, 50, and 70 mg/kg] to assess decursinol-induced antinociception and hypothermia. Mice were assessed via the hot plate and tail-flick assays and for body temperature prior to and 30 min after each decursinol injection. Cumulative dosing of decursinol was performed by giving an IP injection of 10 mg/kg decursinol and testing 30 min later. Immediately after testing, an IP injection of 20 mg/kg decursinol was given to achieve a cumulative dose of 30 mg/kg that was tested 30 min afterwards. Additional cumulative doses of 50 mg/kg decursinol (injection of 20 mg/kg) and 70 mg/kg decursinol (injection of 20 mg/kg) were given, with the effects of each dose tested 30 min later.

#### CENP dose-response

A dose-response curve for decursinol [VEH (0), 1, 3, 10, 30, and 50 mg/kg] was generated in neuropathic mice. Mice were assessed once-daily for 6 (N = 30) consecutive days at increasing concentration of decursinol for mechanical allodynia (the amount of force in grams required to elicit a paw withdrawal response) and body temperature prior to and 30 min after drug administration.

### Daily testing of the acute antinociceptive and hypothermic effects of decursinol to assess tolerance

To determine whether tolerance develops to the antinociceptive and/or hypothermic effects of decursinol, mice were assessed once-daily to either decursinol (50 mg/kg) or vehicle across 14 consecutive days. Mice were baselined for hot plate and tail-flick antinociception and body temperature immediately prior to administration of either 50 mg/kg of decursinol (N = 12) or an equal volume of vehicle (N = 12). Thirty minutes following drug administration, mice were reassessed for hot plate and tail-flick antinociception and for changes in body temperature. Results were reported in %MPE for hot plate and tail-flick and %ΔBT for hypothermia as described above.

### Effects of serotonin receptor (5HT) antagonists on decursinol-induced antinociception

To probe the receptor mechanism of action for decursinol, male mice (N = 80) were given IP injections of vehicle or receptor antagonists 30 min prior to IP injection of vehicle (VEH) or 50 mg/kg decursinol (DOH). The following serotonin antagonists were used: the non-selective 5-HT_2_ receptor antagonist methysergide (MS; 4 mg/kg); the selective 5-HT_2A_ receptor antagonist volinanserin (VOL; 1 mg/kg), and the selective 5-HT_2C_ receptor antagonist SB-242084 (SB; 1 mg/kg). Tail-flick and hot plate antinociception and body temperature were assessed prior to and 30 min after the final injection of vehicle or decursinol resulting in the following 8 groups with 10 mice/group: VEH/VEH; MS/VEH; VOL/VEH; SB/VEH; VEH/DOH; MS/DOH; VOL/DOH; SB/DOH. The dose of methysergide (4 mg/kg) used was chosen based on work from Choi and colleagues (2003b) that found that 4 mg/kg of methysergide was able to attenuate decursinol-induced antinociception in the tail-flick assay. Likewise, 1 mg/kg of SB-242084 was used based on findings that this dose could induce anti-depressant activity (Opal et al., 2014), and a dose of 1 mg/kg volinanserin (MDL100907) was chosen based on studies showing this dose blocked both PCP-enhanced immobility (Corbett et al., 1999) and OSU6162-induced locomotor stimulation (Carlsson et al., 2011) in mice.

### Effects of cannabinoid and opioid receptor antagonists on decursinol-induced anti-allodynia

In this experiment, a separate group of naïve neuropathic mice (N = 13) were tested using cannabinoid receptor type-1 (CB_1_) and type-2 (CB_2_), and opioid receptor antagonists to determine whether the effects of decursinol were mediated through cannabinoid or opioid receptor signaling. Mice were baselined for mechanical allodynia prior to (PRE BL) and following (POST BL) weekly cisplatin treatments to ensure the development of neuropathy. A within-subjects psudo-Latin square design was used to test the effects of vehicle (VEH), 10 mg/kg of the CB_1_ inverse agonist rimonabant (CB_1_A); 10 mg/kg of the CB_2_ inverse agonist, SR144528 (CB_2_A), or 10 mg/kg of the nonselective opioid antagonist naloxone (NXO) alone and in combination with 50 mg/kg of decursinol (DOH). Assignment of the order of drug treatments was randomly selected. Baseline measurements of mechanical allodynia were assessed prior to injection and 30 min after injection of all compounds resulting in the following 8 testing groups: VEH/VEH; CB_1_A/VEH; CB_2_A/VEH; NXO/VEH; VEH/DOH; CB_1_A/DOH; CB_2_A/DOH; NXO/DOH. Doses of cannabinoid inverse agonists were selected based on previous studies showing that 10 mg/kg could block cannabinoid-induced anti-allodynia in neuropathic mice (Henderson-Redmond et al., 2021). Given that Choi and colleagues (2003b) did not find an effect of naloxone on decursinol-induced antinociception following treatment with 4 mg/kg of naloxone, 10 mg/kg of naloxone was used in this experiment to determine if a higher dose of naloxone could negate the effects of decursinol.

### Decursinol-induced ataxia

Motor performance was assessed using a single-lane rotarod (Med-Associates, St. Albans, VT) set to an accelerating rate of 4–40 rpm over 300 s (Marcus et al., 2017). Mice (N = 20) were given six training trials with a duration of 300 s per trial. On testing days, decursinol-induced ataxia was measured in triplicate and averaged 30 min post injection of decursinol. The effects of 0, 3, 10, 20, 30, 40, and 50 mg/kg decursinol were examined with mice allowed at least 4 days of recovery between each dose of decursinol. To determine whether tolerance develops to decursinol-induced ataxia, a separate group of mice was administered once-daily IP injections of 50 mg/kg decursinol (N = 8) or vehicle (N = 2) across 14 consecutive days. Prior to testing, mice were acclimated to the single lane rotarod as described above. On testing days (1, 7, and 14) mice were assessed for their performance on the rotarod 30 min following drug administration and the mean latency to fall was measured in triplicated with a duration maximum of up to 300s per trial. The mean latency was averaged per treatment to identify changes in motor functioning.

### Data analysis

All animals were randomly assigned to treatment groups for all experimental conditions. Group sizes were selected both based on power analysis (G* Power, Dusseldorf, Germany) and previous studies (Nealon et al., 2019; Henderson-Redmond et al., 2021). A power analysis (power = 0.8) revealed minimum group sizes of 10 mice/group to detect a 0.25 difference in means (alpha = 0.05). Given that most experiments will involve in induction of neuropathic pain, sample sizes of ∼12 mice will be used to account for 1-2 that may not meet criteria for the resulting neuropathy (at least a 40% reduction in pain score). Experiments with larger Ns include those that were replicated following relocation of our lab from Penn State to Marshall to ensure consistency in our findings. For all tests, the data generated were expressed as mean ± the standard error of the mean (SEM). Decursinol dose-response data for acute nociception and hypothermia, in neuropathic mice, and the effects of cannabinoid and opioid signaling on decursinol-induced anti-allodynia were all analyzed with repeated measures one-way analysis of variance (ANOVA). Serotonin receptor signaling on decursinol-induced antinociception was analyzed with an ordinary one-way ANOVA and all other assays were assessed using two-way mixed ANOVAs with drug (DOH versus VEH) as the between-subjects factor and day/time as the within-subjects factor. The effective doses (ED_50_) for dose-response data were determined by non-linear regression analyses generated from the dose-response curves and slopes for all tolerance studies were determined using linear regression analyses. All analyses were performed using GraphPad Prism 9.1 (GraphPad Software, Inc. La Jolla, CA). Bonferroni post hoc analyses were performed when appropriate. In all cases significance was set at *p* < 0.05.

## Results

### Decursinol induced acute thermal antinociception and hypothermia

**FIGURE 2:**
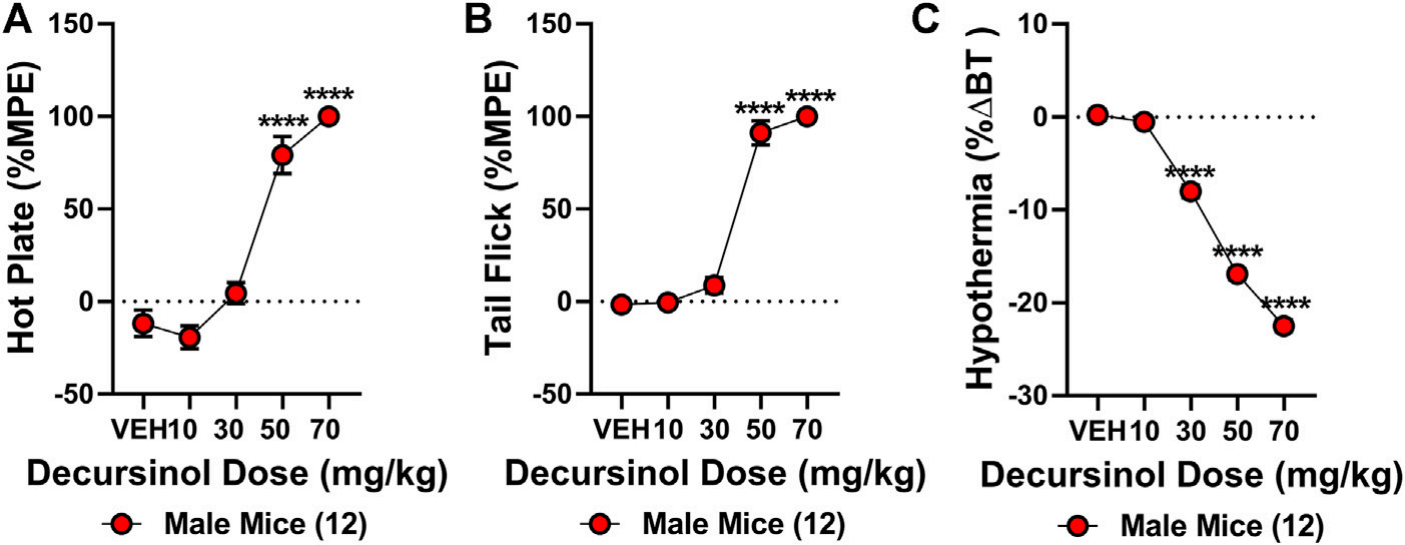
Decursinol-induced antinociception and hypothermia. Cumulative dose-response curves were generated using male mice (N = 12) to assess the antinociceptive and hypothermic effects of vehicle (VEH), 10, 30, 50, and 70 mg/kg decursinol in the hot plate **(A)**, tail-flick **(B)**, and hypothermia **(C)** assays. Mice were injected with increasing doses of decursinol and assessed for antinociception (%MPE) and hypothermia (%ΔBT) 30 min later. Error bars represent the mean *±* SEM. Data were analyzed using repeated measures (RM) one-way ANOVAs and Bonferroni post hoc tests. (****p < 0.0001 compared to VEH).

The antinociceptive effects of decursinol were assessed using a cumulative dose-response curve (0, 10, 30, 50, and 70 mg/kg; IP) in acute models of thermal pain (hot plate and tail-flick assays) and/or hypothermia. One-way ANOVA revealed that decursinol induced antinociception in both the hot plate (*F*_4,44_ = 76.88, *p* < 0.0001) and tail-flick (*F*_4,44_ = 199.6, *p* < 0.0001; Figures 2A,B) assays. Bonferroni post-hoc analyses revealed that for both hot plate and tail-flick assays, neither 10 nor 30 mg/kg of decursinol was able to induce antinociception. However, doses of 50 (*p* < 0.0001) and 70 (*p* < 0.0001) mg/kg were able to induce significant antinociception in both assays. Given that administration of 70 mg/kg did not induce a significantly greater increase in decursinol-mediated antinociception in either assay, 50 mg/kg of decursinol was used for all subsequent studies examining decursinol as a potential therapeutic for pain management. ED_50s_ for both hot plate and tail-flick were generated using their dose-response curves using non-linear regression analyses (Table 1). As opioids induce hypothermia, we also assessed whether decursinol had hypothermic effects. One-way ANOVA indicated that decursinol dose-dependently induced hypothermia (*F*_4,44_ = 701.9, *p* < 0.0001; Figure 2C). Post-hoc analyses revealed that while there was no change in response following 10 mg/kg of decursinol, administration of all other doses (30, 50, and 70 mg/kg) further increased hypothermic responses compared to vehicle (*p* < 0.0001) and from all other doses tested (*p* < 0.0001). As with hot plate and tail-flick, the ED_50_ value for hypothermia was determined using the dose-response curve generated (Table 1).

**TABLE 1:** Calculated ED_50_ values (mg/kg) for the acute thermal antinociceptive and hypothermic effects of decursinol. ED_50_ values were calculated from cumulative dose-response curves generated using non-linear regression analysis. Values shown are mean ED_50_ dose and 95% confidence intervals for decursinol in mice (N = 12/group). UNK = values that could not be calculated.

Drug		Hot plate	Tail-flick	Hypothermia
Decursinol	ED_50_	39.71	38.63	35.43
—	95% CI	34.16–46.72	UNK-42.43	33.41–37.64

### Tolerance develops to the antinociceptive (but not hypothermic) effects of decursinol

**FIGURE 3:**
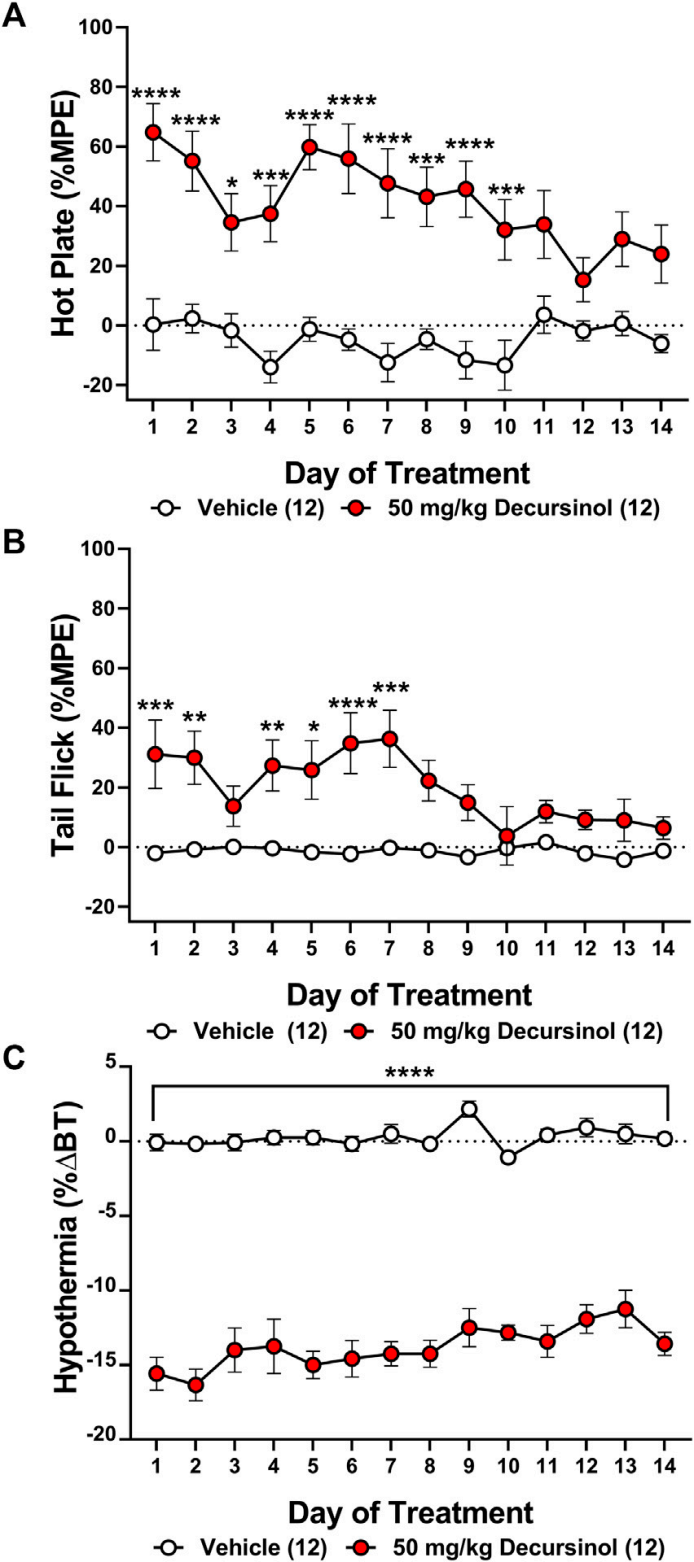
Development of antinociceptive (but not hypothermic) tolerance to once-daily 50 mg/kg of decursinol. Tolerance to the antinociceptive and hypothermic effects of 50 mg/kg of decursinol (filled circles; N = 12) or vehicle (unfilled circles; N = 12) was examined in male mice. Mice were assessed for antinociception via the hot plate **(A)** and tail-flick **(B)** assays and for hypothermia **(C)** following treatment with either decursinol or vehicle (VEH). Data are expressed as mean ± SEM and were analyzed using a two-way ANOVA with Bonferroni post-hoc tests. (*p < 0.05, **p < 0.01, ***p < 0.001, ****p < 0.0001 compared to VEH).

Tolerance to the antinociceptive effects of drugs, including opioids, represents a major barrier to their clinical utility. To date, no literature exists addressing whether tolerance develops for AGN or decursinol following prolonged administration. As such, we assessed whether tolerance develops to the antinociceptive or hypothermic effects of once-daily administration of decursinol (50 mg/kg) in mice. Two-way ANOVAs indicated main effects of treatment (decursinol) in both the hot plate (*F*_1,22_ = 99.33, *p* < 0.0001) and tail-flick (*F*_1,22_ = 28.06, *p* < 0.0001) assays as well as for hypothermia (*F*_1,22_ = 603.1, *p* < 0.0001; Figure 3). There were also main effects of time (day) [hot plate (*F*_13,286_ = 2.290, *p* = 0.0069); tail-flick (*F*_13,286_ = 2.399, *p* = 0.0045); hypothermia (*F*_13,286_ = 2.528, *p* = 0.0027)] with the effects of decursinol decreasing following prolonged administration. Finally, there was a significant treatment-by-time interaction effect for both hot plate (*F*_13,286_ = 1.977*, p* = 0.0225) and tail-flick (*F*_13,286_ = 2.354, *p* = 0.0053) but not for hypothermia (*p* = 0.2302; Figure 3).

Bonferroni post-hoc tests were utilized to assess when “full tolerance” [day(s) where there is not a significant difference in response between vehicle and decursinol] developed to decursinol. Tolerance developed on day 11 for hot plate (*p* = 0.1063), and day 8 for tail-flick (*p* = 0.0573; Figures 3A,B). In contrast, mice failed to develop full tolerance to the hypothermic effects of decursinol following 14 days of once-daily treatment (Figure 3C). As tolerance development is progressive and occurs over time, to account for the fluctuation of responses over day, we calculated the slope of the regression line over time to determine the rate of tolerance (Table 2). Using the slopes, we saw tolerance was faster to develop to the antinociceptive (hot plate: -2.618; tail-flick: -1.944) versus hypothermic (0.2639) effects of decursinol.

**TABLE 2:** Calculated slopes (mean +95% confidence intervals) and x-intercepts for the acute antinociceptive and hypothermic effects of vehicle (VEH)- and decursinol (DOH)-treated groups across day. Linear regression analyses were used to determine the slopes from the daily tolerance curves following once-daily treatment with either vehicle (VEH) or decursinol (DOH) across 14 days. The slopes were extrapolated to determine the *x*-intercept indicative of when the response following treatment would be 0. Values shown are the slopes and extrapolated x-intercepts and 95% confidence intervals for vehicle and decursinol treated mice (N = 12/group). ∞ = values that could not be calculated/were listed as infinity. Significance denotes a significant difference in the slope from 0.

Assay	Drug	Slope	X-intercept	Significance
		Mean (CI)	Mean (CI)	
Hot Plate	VEH	−0.1176 (^-^4.020- -1.216)	−31.71 (^-^∞-4.917)	*p* = 0.7807
—	DOH	−2.6180 (^-^1.018–0.0782)	23.28 (17.51–41.75)	*p* = 0.0016**
Tail flick	VEH	−0.0686 (^-^0.287–0.150)	−11.34 (^-^∞-4.556)	*p* = 0.5069
—	DOH	−1.9440 (^-^3.093–0.796)	17.66 (13.43–32.79)	*p* = 0.0031**
Body Temperature	VEH	0.0449 (^-^0.0601–0.1499)	2.061 (^-^∞-∞)	*p* = 0.3701
—	DOH	0.2639 (0.138–0.390)	59.8 (42.83–107.6)	*p* = 0.006**

### 5-HT_2_ signaling enhances decursinol-induced antinociception and hypothermia

**FIGURE 4:**
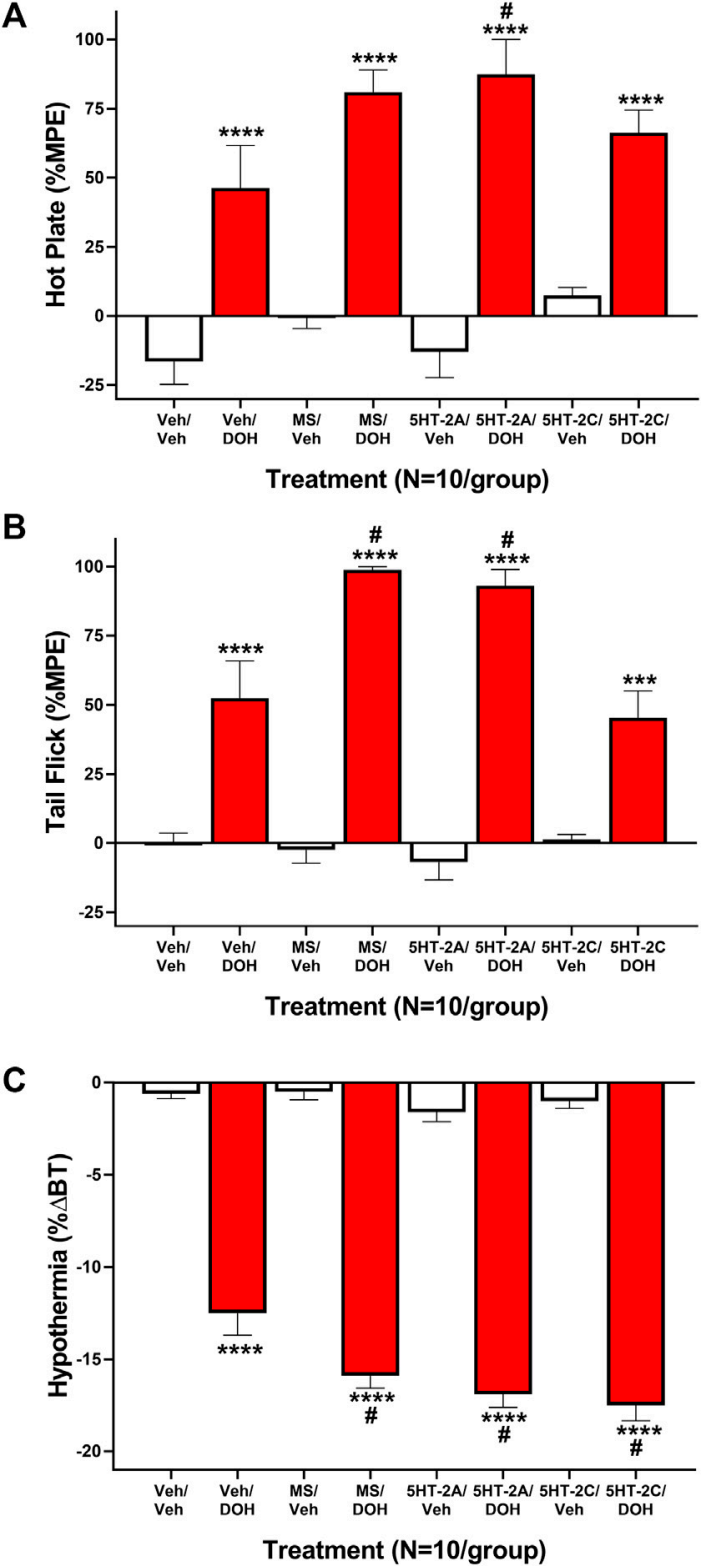
Serotonin Receptor 2 (5-HT_2_) signaling does not attenuate decursinol-induced antinociception or hypothermia. Mediation of the acute antinociceptive (A/B) and hypothermic **(C)** effects of 50 mg/kg decursinol by 5-HT_*2*_ antagonists in male mice. Mice (N = 10) were assessed for hot plate **(A)** and tail-flick **(B)** antinociception (%MPE) and hypothermia (C; %ΔBT) 30 min following pretreatment with either vehicle (VEH), 4 mg/kg of the non-selective 5-HT_2_ antagonist Methysergide (MS), 4 mg/kg of the selective 5-HT_2A_ antagonist Volinanserin (5-HT_2A_), or 4 mg/kg of the selective 5-HT_2C_ receptor antagonist SB-242084 (5-HT_2C_) and again 30 min following treatment with either vehicle (VEH; unfilled bars) or 50 mg/kg of decursinol (DOH; filled bars). Error bars represents the mean ± SEM. Data were analyzed using a repeated measures one-way ANOVA with Bonferroni post-hoc tests (***p < 0.001; ****p < 0.0001 compared to VEH/VEH; ^*#*^ *p* < 0.05 compared to VEH/DOH).

Previous research suggests that the acute antinociceptive effects of decursinol may be partially mediated via the serotonin 2 (5-HT_2_) receptor subtype (Choi et al., 2003b). As such, we assessed nociception following treatment with selective and non-selective 5-HT_2_ antagonists both alone and in combination with decursinol. One-way ANOVA revealed main effects of treatment for both hot plate (*F*_7,72_ = 20.94, *p* < 0.0001) and tail-flick (*F*_7,72_ = 39.52, *p* < 0.0001) (Figures 4A,B). Post-hoc analyses revealed that for both hot plate and tail-flick, none of the serotonin antagonist utilized [the nonselective 5-HT_2_ antagonist methysergide (MS); the selective 5-HT_2A_ antagonist volinanserin (5-HT_2A_), and the 5-HT_2C_ selective antagonist SB-242084 (5-HT_2C_)] differed from vehicle (VEH) when given alone. Surprisingly, none of the selective antagonists attenuated the antinociceptive effects of decursinol. And contrary to published data, pretreatment with MS enhanced decursinol-mediated antinociception in the tail-flick test (*p* < 0.0001). This effect was likely mediated through the 5-HT_2A_ receptor subtype as pretreatment with the selective 5-HT_2A_ (but not 5-HT_2C_) antagonist also further enhanced decursinol-mediated antinociception in both the hot plate (*p* = 0.0106; Figure 4A) and tail-flick (*p* = 0.0004 Figure 4B) assays. There was also a main effect of treatment on hypothermia (*F*_7,72_ = 136.8, *p*=<0.0001); MS (*p* = 0.0057), 5-HT_2A_ (*p* = 0.0002), and 5-HT_2C_ (*p* < 0.0001) pretreatment all enhanced decursinol-induced hypothermia (Figure 4C).

### Decursinol dose-dependently reverses allodynia in neuropathic mice

**FIGURE 5:**
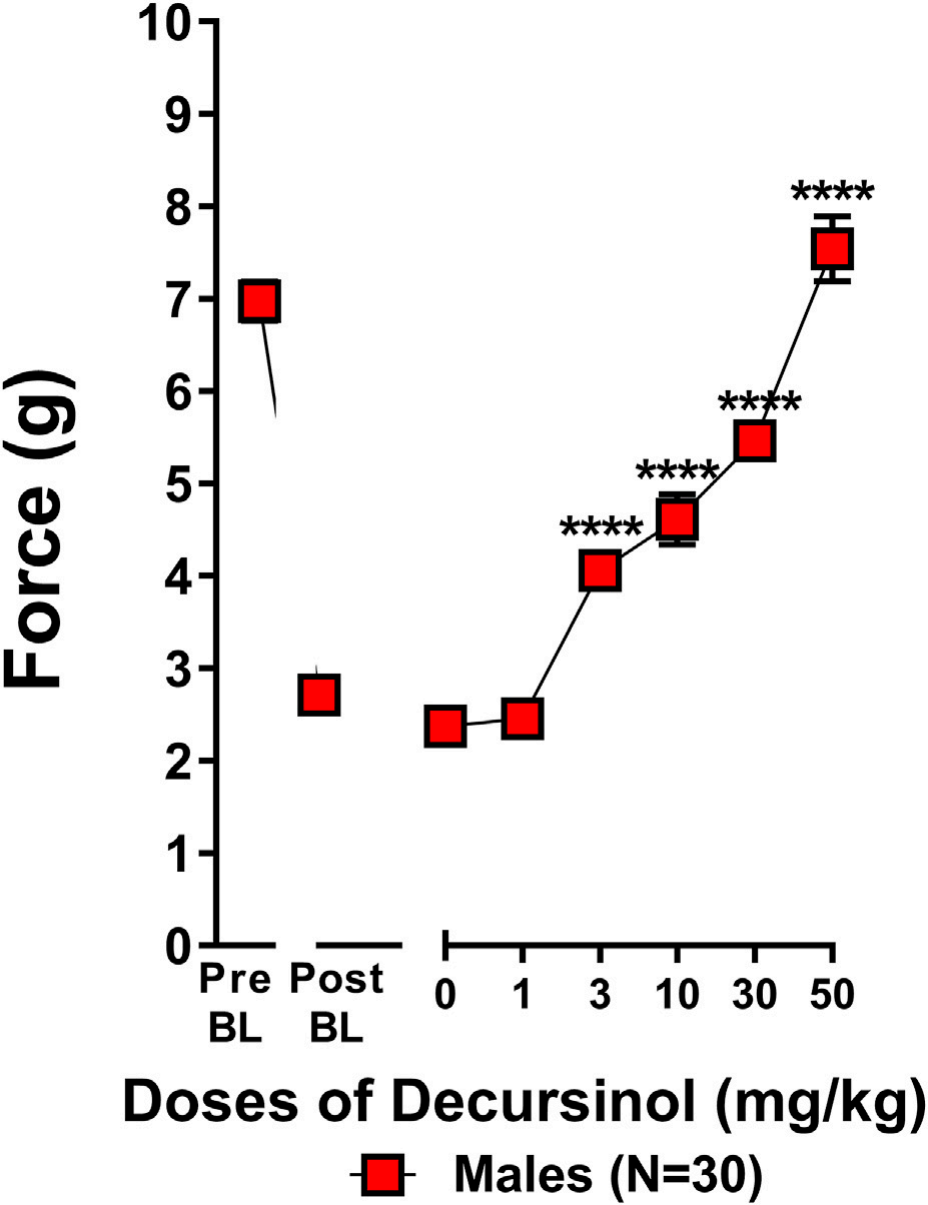
Decursinol dose-dependently reverses cisplatin-induced mechanical allodynia in neuropathic male mice. Dose-response curves were generated using male mice to assess the anti-allodynic effects of decursinol in mice with cisplatin-induced neuropathy. Mice were assessed via the von Frey for the amount of force (in grams) required to elicit a paw withdrawal response prior to cisplatin treatment (Pre BL), following cisplatin-evoked neuropathy (Post BL), and 30 min following treatment with either vehicle (0) and 1, 3, 10, 30, and 50 mg/kg decursinol (DOH; N = 30). Error bars represents the mean ± SEM. Each mouse was tested in triplicate per dose, and these values were averaged into a single value per mouse per day of testing. Data were analyzed using a repeated-measures one-way ANOVA with Bonferroni post-hoc tests. (****p < 0.001 compared to 0).

A dose-response curve was generated to determine which dose(s) of decursinol (0, 1, 3, 10, 30, and 50 mg/kg) were capable of reversing allodynia (Figure 5). An initial t-test confirmed that cisplatin induced neuropathy (allodynia; *t*_29_ = 17.80, *p* < 0.0001). One-way ANOVA revealed that decursinol (*F*_5,145_ = 88.48, *p* < 0.0001) dose-dependently reversed allodynia. The ED_50_ value for decursinol was calculated using non-linear regression analyses from the dose-response curve (Table 3). Bonferroni post-hoc analyses revealed that all doses of decursinol except for 1 mg/kg were able to at least partially reverse allodynia and that only 50 mg/kg was able to fully reverse allodynia to pre-baseline levels. Doses of 30 and 50 mg/kg decursinol incrementally induced greater anti-allodynic responses (Figure 5) in neuropathic mice.

**TABLE 3:** Calculated ED_50_ value (mg/kg) assessing the anti-allodynic effect of decursinol in neuropathic mice. The ED_50_ value was calculated from a dose-response curve generated using non-linear regression analysis. The value shown is the mean ED_50_ dose and 95% confidence interval for decursinol in neuropathic mice (N = 30). UNK = value(s) that could not be calculated.

Drug		Reversal of mechanical allodynia
Decursinol	ED_50_	10.75
—	95% CI	7.816-UNK

### Tolerance to the anti-allodynic and hypothermic effects of decursinol in neuropathic mice

**FIGURE 6:**
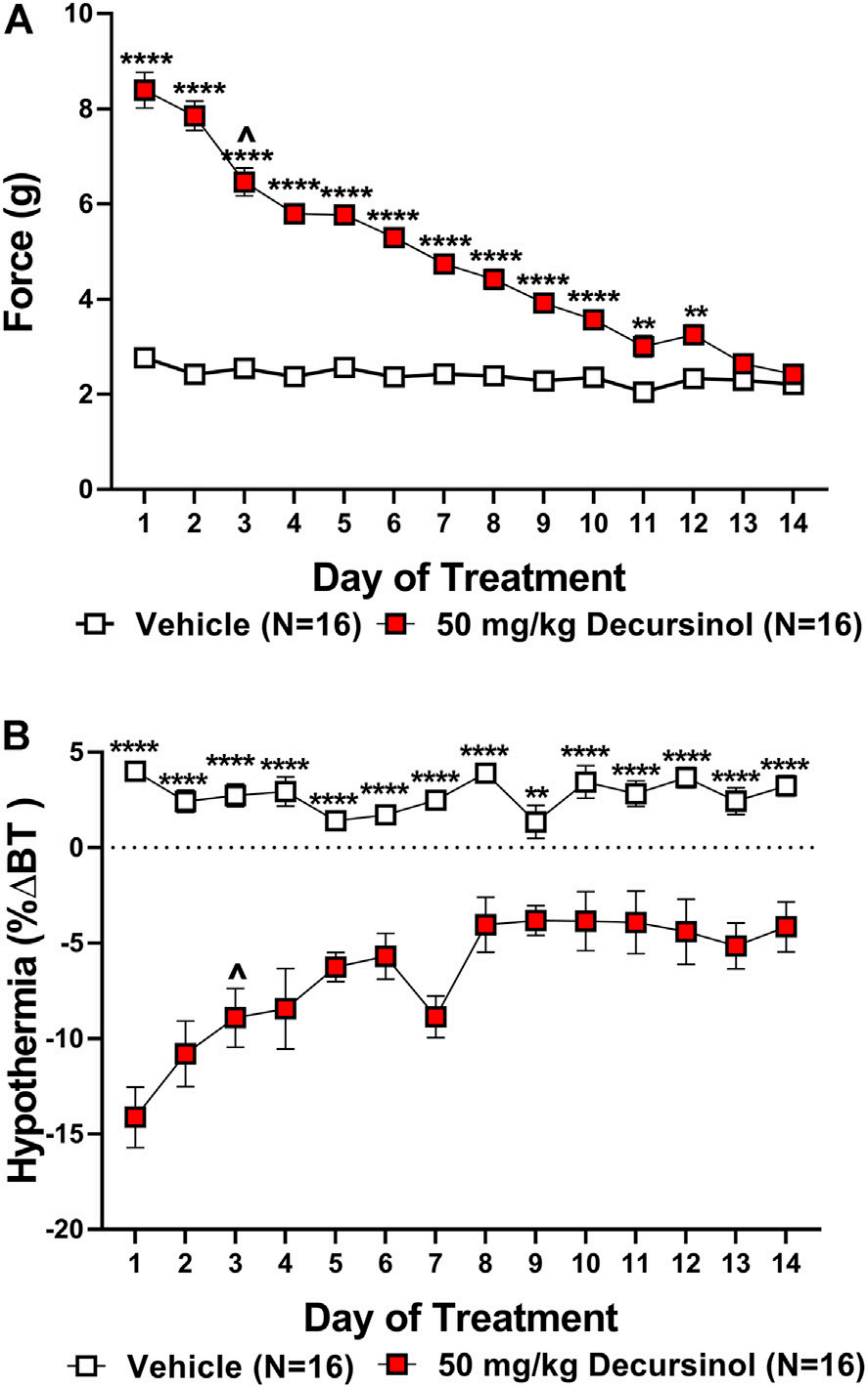
Development of tolerance to the anti-allodynic and hypothermic effects of once-daily administration of 50 mg/kg decursinol in neuropathic mice. Tolerance to the anti-allodynic **(A)** and hypothermic **(B)** effects of decursinol in neuropathic mice was assessed 30 min following once-daily administration of either vehicle (open squares; N = 16) or 50 mg/kg of decursinol (filled squares; N = 16). Mice were assessed for the amount of force (in grams) required to elicit a paw withdrawal response (30 min) and body temperature (40 min) following treatment with either decursinol (DOH; 50 mg/kg) or vehicle (VEH). Error bars represents the mean ± SEM. Each moue was tested in triplicate and those values averaged into a single value per mouse per day of treatment. Data were analyzed using a two-way ANOVA with Bonferroni post-hoc tests (*p < 0.05, **p < 0.01, ***p < 0.001, ****p < 0.0001; ^*p* < 0.05 compared to Day 1 of DOH treatment indicative of the first day of partial tolerance development).

As with acute antinociception, we assessed how tolerance develops to the anti-allodynic effects of decursinol in neuropathic mice. Two-way ANOVAs indicated main effects of treatment (decursinol) for both mechanical allodynia (*F*_1,30_ = 653.9, *p* < 0.0001) and hypothermia (*F*_1,30_ = 91.50, *p* < 0.0001; Figure 6). There were main effects of time (day) [mechanical allodynia (*F*_13,390_ = 62.87, *p* < 0.0001); hypothermia (*F*_13,390_ = 6.210 *p* < 0.0001)] with the effects of decursinol decreasing across 14 days of treatment. Finally, there was a significant treatment-by-time interaction effect for both mechanical allodynia (*F*_13,390_ = 46.96*, p* < 0.0001) and hypothermia (*F*_13,390_ = 7.210, *p* < 0.0001; Figure 6). Bonferroni post-hoc tests revealed that by day 13, mice were fully tolerant to the anti-allodynic effects (Figure 6A) but that full tolerance did not develop to the hypothermic effects of decursinol in neuropathic mice after 14 days of treatment (Figure 6B). Linear regression analyses estimating the rate of tolerance development found tolerance development was faster to the anti-allodynic (-4.370) versus the hypothermic effects of decursinol (0.6299) in neuropathic mice (Table 4).

**TABLE 4:** Calculated slopes (mean +95% confidence intervals) and x-intercepts for the anti-allodynic and hypothermic effects of vehicle (VEH)- and decursinol (DOH)-treated groups across day in neuropathic mice. Linear regression analyses were used to determine the slopes from the daily tolerance curves following once-daily treatment with either vehicle (VEH) or decursinol (DOH) across 14 days of once-daily treatment in mice with neuropathic pain. The slopes were extrapolated to determine the *x*-intercept indicative of when the response following treatment would be 0. Values shown are the slopes and extrapolated x-intercepts and 95% confidence intervals for vehicle and decursinol treated mice (N = 12/group). ∞ = values that could not be calculated/were listed as infinity. Significance denotes a significant difference in the slope from 0.

Assay	Drug	Slope	X-intercept	Significance
		Mean (CI)	Mean (CI)	
Von Frey	VEH	−0.03109 (^-^0.0476- ^-^0.0146)	84.08 (57.48–170.6)	*p* = 0.0015**
—	DOH	−4.370 (^-^4.973–3.765)	18.54 (17.1–20.4)	*p <* 0.0001****
Body Temperature	VEH	0.01823 (^-^0.1102–0.1467)	-143.0 (^-^∞-^-^10.88)	*p* = 0.7625
—	DOH	0.6299 (0.3638–0.8960)	17.99 (14.55–25.98)	*p* = 0.0002***

### Anti-allodynic effect of decursinol was partially attenuated by opioid antagonism

**FIGURE 7:**
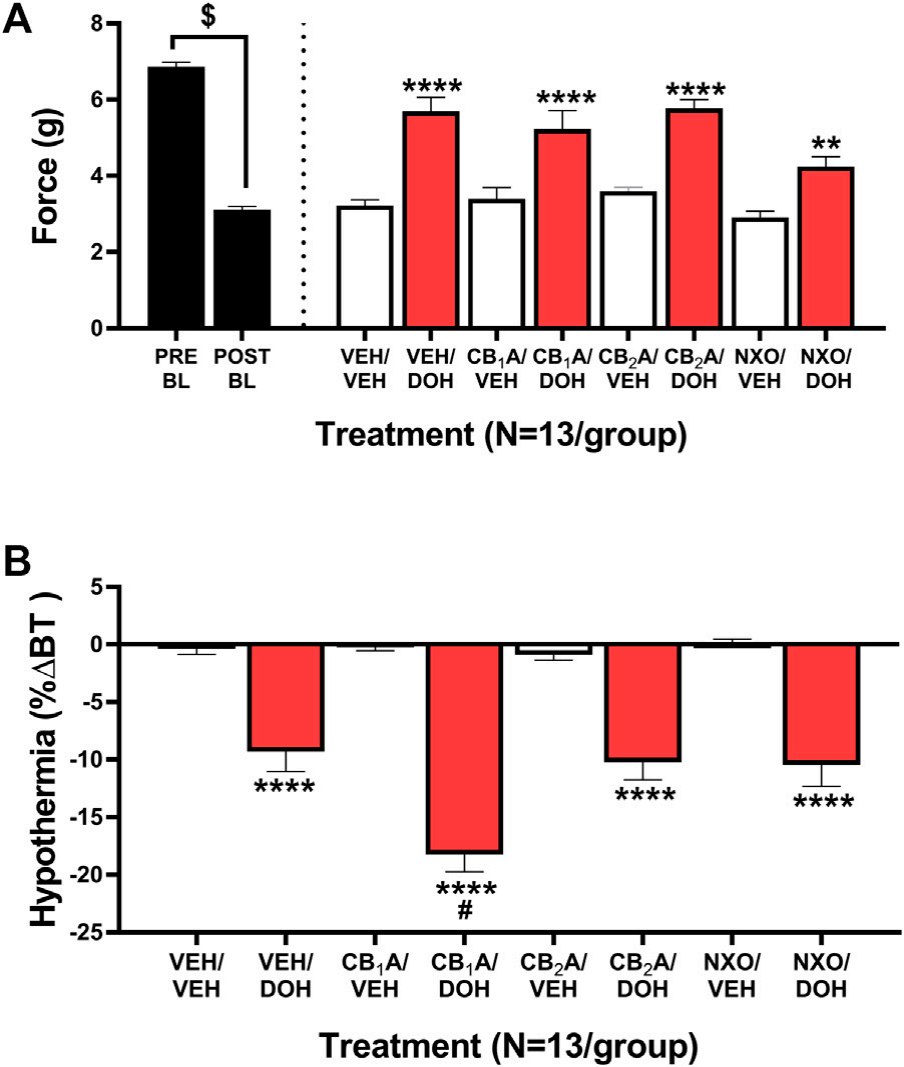
Cannabinoid and opioid mediation of the anti-allodynic and hypothermic effects of decursinol in mice with cisplatin-induced neuropathy. Mediation of the anti-allodynic **(A)** and hypothermic **(B)** effects of 50 mg/kg decursinol by CB_1_, CB_2_, and/or opioid receptors in male mice (N = 13). Mice were assessed via the von Frey for the amount of force (in grams) required to elicit a paw withdrawal response prior to cisplatin treatment (Pre BL) and following cisplatin-evoked neuropathy (Post BL). Mice were also assessed for the ability of the CB_1_ inverse agonist Rimonabant (CB_1_A), the CB_2_ inverse agonist SR144528 (CB_2_A), and the nonselective opioid antagonist naloxone (NXO) to mediate the anti-allodynic effects of 50 mg/kg of decursinol by administering either 10 mg/kg of CB_1_A or CB_2_A, 10 mg/kg of NXO, or vehicle (VEH) 30 min prior to treatment with either decursinol (DOH) or vehicle (VEH). For each mouse, testing occurred in triplicate per mouse and was averaged into a single value per mouse per day of testing. Error bars represents the mean ± SEM. Data were analyzed using a repeated measures one-way ANOVA with Bonferroni post-hoc tests (**p < 0.01,****p < 0.0001 compared to VEH/VEH; ^*#*^ *p* < 0.05 compared to VEH/DOH; ^$^ *p* < 0.0001 Pre versus Post BL).

Despite potential roles for the adrenergic, serotonergic, and opioid systems in modulating decursinol antinociception in acute pain model (Choi et al., 2003b), the role of cannabinoid and opioid signaling has yet to be investigated in decursinol-mediated anti-allodynia. As such, neuropathic mice were assessed to determine if the anti-allodynic and/or hypothermic effects of decursinol (50 mg/kg) were mediated by cannabinoid and/or opioid receptors. An initial t-test revealed that cisplatin induced neuropathy (allodynia; *t*_12_ = 28.62, *p* < 0.0001). One-way ANOVA indicated main effects of treatment for the anti-allodynic (*F*_7,84_ = 16.54, *p* < 0.0001) and hypothermic effects (*F*_7,86_ = 37.06, *p* < 0.0001) of decursinol (Figure 7). Post-hoc analyses revealed that pretreatment with the CB_1_ inverse agonist rimonabant (CB_1_A), the CB_2_ inverse agonist SR144528 (CB_2_A), and the nonselective opioid antagonist naloxone (NXO) alone did not differ from vehicle. In contrast, pretreatment with NXO at 10 mg/kg, (but not CB_1_A or CB_2_A) was able to partially block the anti-allodynic effects of decursinol in neuropathic mice (*p* = 0.0010), suggesting that opioid receptor signaling may mediate decursinol-induced anti-allodynia (Figure 7A). In contrast, while pretreatment with CB_2_A and NXO failed to have an effect, pretreatment with CB_1_A enhanced decursinol-induced hypothermia (*p* < 0.0001; Figure 7B).

### Decursinol dose-dependently induced motor impairment on the rotarod

**FIGURE 8:**
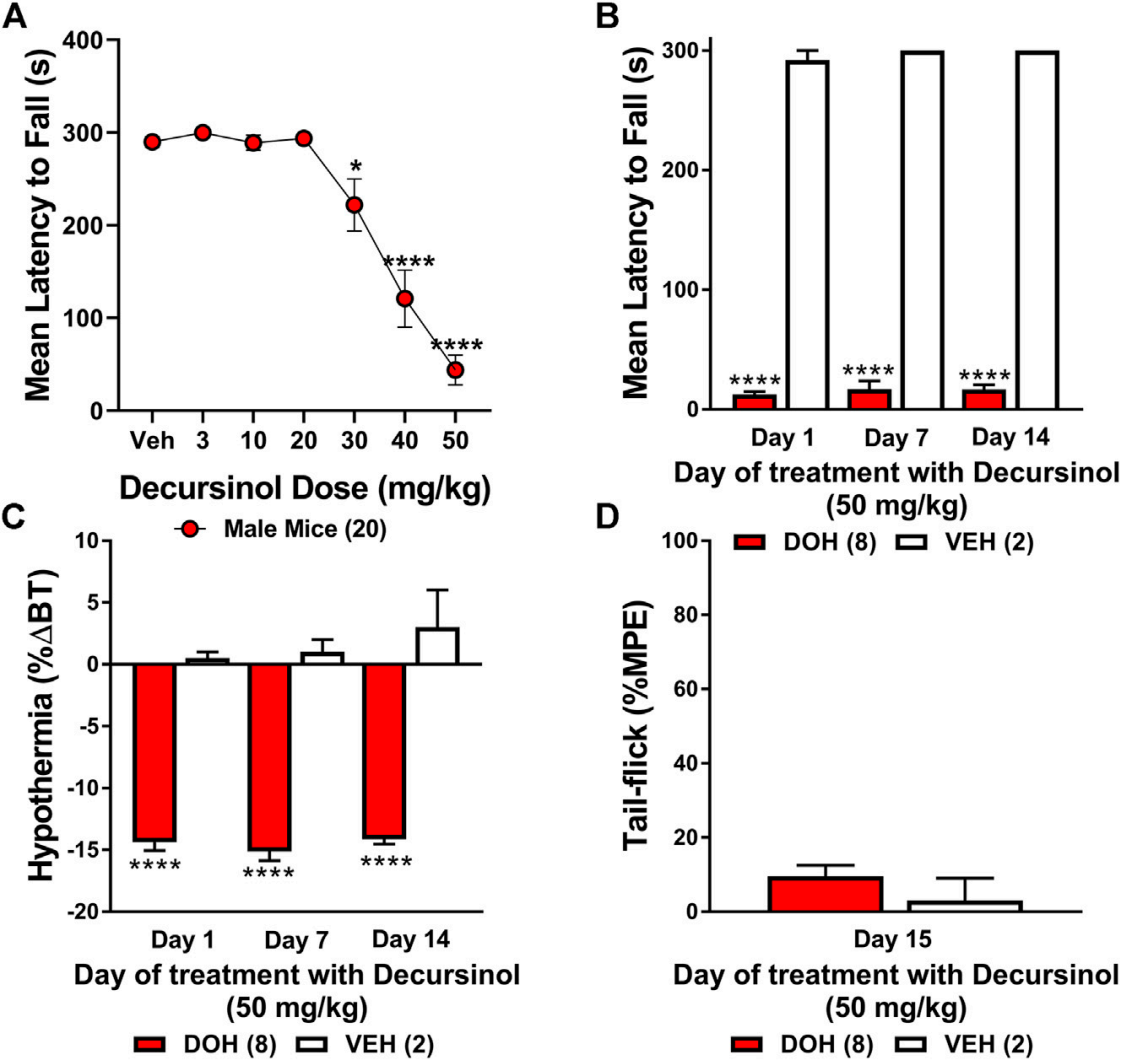
Decursinol-induced ataxia in male mice. Mice were assessed for the ataxic effects of decursinol **(A)** across a range of doses and tolerance to both the ataxic **(B)** and hypothermic **(C)** effects of decursinol to dissociate confounds of ataxia on tolerance to the antinociceptive effects of decursinol on tail-flick **(D)**. Using an accelerating rotarod, mice were assessed for the mean latency (in seconds) to fall 30 min post decursinol administration on the rotarod across dose **(A)** or on days 1, 7, and 14 across 14 days of once-daily administration of 50 mg/kg decursinol (N = 8; red bars) or vehicle (VEH; N = 2; white bars). Mice were also assessed for tolerance to hypothermia on days 1, 7, and 14 of decursinol administration **(C)** and on Day 15 to see if mice were tolerant to the antinociceptive effects of decursinol following 14 days of once-daily administration on the tail-flick. Error bars represents the mean ± SEM and data were analyzed using a repeated measure one-way **(A)** or two-way **(B,C)** ANOVA with Bonferroni post-hoc tests (*p < 0.05, ****p < 0.0001 compared to Veh) or a t-test **(D)**.

While assessing tolerance to the anti-allodynic effects of decursinol in neuropathic mice, we observed a profound sedative effect, leading us to test whether decursinol might exert dose-dependent effects on ataxia using the rotarod test (Figure 8A). One-way ANOVA revealed that mice exhibited a dose-dependent increase in decursinol-induced ataxia (*F*_6,114_ = 38.12, *p* < 0.0001). Post-hoc analyses revealed that decursinol-induced ataxia was observed following treatment with 30 (*p* = 0.0256), 40 (*p* < 0.0001), and 50 (*p* < 0.0001) mg/kg of decursinol with increasing doses further increasing the severity of motor impairment. Given that the dose of decursinol utilized in our studies (50 mg/kg) can cause ataxia, we sought to determine whether tolerance to the ataxic effects of decursinol might confound our findings regarding tolerance to the pain-relieving effects of decursinol. Results from a two-way ANOVA revealed a significant main effect of drug treatment (*F*_1,8_ = 2,390, *p* < 0.0001) but not a day (*p* = 0.6321) or a drug-by-day interaction (*p* = 0.9592). The results revealed that mice treated with decursinol (50 mg/kg) once-daily showed significant motor impairment across all testing (1, 7, and 14) days compared to mice treatment with vehicle (*p* < 0.0001) once-daily. Further, on day 14, mice were just as impaired on the rotarod as on day 1 of testing, suggesting that tolerance to the antinociceptive and/or anti-allodynic effects of decursinol occurred independent of decursinol-induced ataxia (Figure 8B). As in our non-neuropathic mice, decursinol induced significant hypothermia (main effect of drug *F*_1,8_ = 242.2, *p* < 0.0001) compared to vehicle-treated mice and there was no evidence of tolerance to decursinol-induced hypothermia (*p* = 0.2395; Figure 8C). Finally, mice tested on the tail-flick assay on day 15 did not differ from vehicle mice in their antinociceptive response (*p* = 0.3466) and showed evidence of complete antinociceptive tolerance (Figure 8D).

## Discussion

The primary aims of this study were to assess whether tolerance develops to the antinociceptive effects of once-daily administered decursinol (50 mg/kg; IP) in acute thermal pain models, 2) to establish its anti-allodynic efficacy and potential tolerance development in a model of chemotherapy-evoked neuropathic (CENP) pain, and 3) to probe the involvement of select receptors in mediating the pain-relieving effects with antagonists. We found that decursinol, at 50 mg/kg, induced antinociception in both the hot plate and tail-flick assays and reversed allodynia in a model of cisplatin-evoked neuropathy. In addition, while tolerance was eventually detected to the antinociceptive and anti-allodynic effects of decursinol, mice did not develop tolerance to decursinol-induced hypothermia. Furthermore, contrary to what has previously been reported, 5-HT_2_ receptors did not mediate the effects of acute decursinol-induced antinociception while opioid signaling at least partially mediated decursinol-induced anti-allodynia.

The finding that decursinol was able to elicit antinociception in the hot plate and tail-flick assays was consistent with previous reports in acute pain models, including the hot plate, tail-flick, formalin, (Choi et al., 2003a; 2003b), and writhing (Choi et al., 2003a, 2003b; Seo et al., 2009) tests. Similarly, while this was the first study to evaluate the anti-allodynic properties of decursinol in a model of chemotherapy (cisplatin)-evoked pain, a recent study by Son and colleagues found that decursin, which is metabolized in the liver into decursinol, was able to attenuate mechanical allodynia in a paclitaxel-induced model of neuropathic pain when given intrathecally (2021). Interestingly, the anti-allodynic effects of decursin were only observable following repeat injections of decursin in decursin-treated mice (Son et al., 2021). Whether such a delay was due to the induction of metabolic conversion in the CNS to decursinol is an open question. Nevertheless, our study offers further support that decursinol has analgesic properties in both acute and neuropathic pain models with the novelty of our experiments being the first to assess whether tolerance develops to the analgesic properties of decursinol.

Involvement of alpha (α)2-adrenergic, histamine, and serotonergic signaling had been implicated in decursinol-mediated/induced antinociception (Choi et al., 2003b). Based on such findings, we evaluated whether pretreatment with the non-selective 5-HT_2_ antagonist methysergide, the selective 5-HT_2A_ antagonist volinanserin, or the selective 5-HT_2C_ receptor antagonist SB-242084 could affect decursinol-induced antinociception. In contrast to what Choi and colleagues showed (2003b), we found that blocking 5-HT_2_ signaling with methysergide enhanced rather than blocked decursinol-induced antinociception, and that this effect was likely mediated through 5-HT_2A_ receptors as pretreatment with volinanserin (but not SB-242084) significantly enhanced decursinol-induced antinociception in both the hot plate and tail-flick assays. Likewise, pretreatment with all three drugs further enhanced decursinol-induced hypothermia.

While serotonin has been shown to mediate pain responses (Yaksh and Wilson 1979; Cui et al., 1999), there are inconsistencies in how various serotonin receptor subtypes may influence antinociception. Intrathecal administration of 5-HT_2_ receptor agonists, including DOI, MK-212, and α-methyl-5-HT all dose-dependently induced antinociception through inhibition of the pressor response and dose-dependent elevation of the visceromotor threshold to noxious colorectal distension (CRD) in a model of visceral pain. Intrathecal pretreatment with the 5-HT_2_ antagonist methysergide also blocked DOI- and α-methyl-5-HT-induced antinociception while pretreatment with the 5-HT_2A_ antagonist ketanserin blocked MK-212-induced antinociception (Danzebrink and Gebhart 1991). Intrathecal pretreatment with ketanserin and the 5-HT_2C_ antagonist mesulergine attenuated acetaminophen- (Courade et al., 2001) and serotonin- (Bardin et al., 2000) induced antinociception in the paw pressure assay. Pretreatment with the 5-HT_2C_ antagonist D-MC blocked serotonin-induced antinociception in both phases of the formalin test while use of the 5-HT_2C_ agonist MK-212 suppressed formalin-induced nociception (Jeong et al., 2004). In contrast, ketanserin enhanced imipramine-induced antinociception in rats with CRD-induced visceral pain (Ilkaya et al., 2014), and subcutaneous (SC) administration of ketanserin produced dose-dependent antinociception in the hot plate assay and acetic acid-induced writhing test but did not affect tail-flick antinociception (Alhaider 1991). Unexpectedly, intrathecal administration of methysergide and ketanserin reversed the antinociceptive effects of subcutaneous ketanserin, indicating that 5-HT_2_ receptors located supraspinally may inhibit descending nociceptive transmission while spinal receptors may modulate nociception (Alhaider 1991). Taken together, these results suggest that the route of antagonist administration can determine whether 5-HT_2_ antagonism attenuates and/or enhances decursinol-induced antinociception. While both we and the study by Choi and colleagues (2003b) administered dose(s) of 5-HT_2_ antagonists intraperitoneally, the route (IP versus oral) and doses of decursinol (50 versus 100 mg/kg) utilized varied, which could account for our observed differences in how serotonin signaling may mediate decursinol-induced antinociception.

Decursinol-induced effects of analgesia, hypothermia, and ataxia (hypo locomotion) and observed sedation mimic the tetrad of behaviors induced following cannabinoid administration (Metna-Laurent et al., 2017; Moore and Weerts 2021). Likewise, morphine administration has dose-dependent hypothermic and antinociceptive effects (Nemeroff et al., 1979; Kest et al., 2000; Marcus et al., 2015; Henderson-Redmond et al., 2016; Nealon et al., 2018). This led us to speculate that decursinol might be acting through G_i_/G_o_ G protein-coupled receptors (GPCRs) much like cannabinoids (Slipetz et al., 1995; Rinaldi-Carmona et al., 1996) and opioids (North et al., 1987). Given that most cannabinoids, including delta-9 tetrahydrocannabinol (Δ^9^-THC), CP55,940, and WIN55,212–2, are mixed agonist acting at both the cannabinoid type-1 (CB_1_) and type-2 (CB_2_) receptors, we assessed whether pretreatment with the selective CB_1_ antagonist rimonabant, the selective CB_2_ antagonist SR144528, or the opioid antagonist naloxone could prevent decursinol-induced anti-allodynia in a model of neuropathic pain. Interestingly, despite the similarities in behavioral responses induced by decursinol, we found that the anti-allodynic effects of decursinol were not mediated *via* cannabinoid signaling. Surprisingly, we did find that pretreatment with rimonabant enhanced decursinol-induced hypothermia. CB_1_-induced hypothermia has been reported to occur *via* CB_1_-stimulated GABA release in the hypothalamus (Rawls et al., 2004). Likewise, administration of the decursinol precursor decursinol angelate has been shown to potentiate pentobarbital-induced sleeping behaviors through the activation of the GABA_A_-ergic systems (Woo et al., 2017). Therefore, it is possible that CB_1_ receptors play a role in modulating decursinol-induced hypothermia, possibly through GABA_A_ activation.

We found that pretreatment with naloxone partially prevented decursinol-reversal of allodynia (but not hypothermia), indicating that opioid signaling may at least partially be responsible for decursinol-induced anti-allodynia. This contrasts with findings by Choi et al., 2003b showing naloxone pretreatment did not prevent decursinol-induced antinociception in the tail-flick assay. However, Choi used only 4 mg/kg of naloxone (versus our 10 mg/kg) which may not have been a strong enough dose to reverse antinociception. For example, pretreatment with 10 mg/kg (but not 0.63 or 2.5 mg/kg) of naloxone was able to partially mediate fentanyl-induced hypothermia in NMRI mice (Baker and Meert 2002). Naloxone, typically deemed a nonselective opioid antagonist, has been reported to be more selective for the mu-opioid receptor, usually at lower doses. Thus, it is possible that doses of 10 mg/kg may be more likely to block delta- and/or kappa-opioid receptors than lower doses, suggesting that our effects might be mediated through delta and/or kappa as opposed to mu-opioid receptors. Son and colleagues also investigated whether decursin could modulate capsaicin-induced rises of intracellular CA^2+^ levels, a hallmark of neuropathic pain in F11 cells and found that decursin could be exerting its effects through inhibition of the transient receptor potential vanilloid 1 (TRPV1) as a potent antagonist (2021). Future studies should examine whether the anti-allodynic effects of decursinol are mediated *via* TRPV1 given that TRPV1 activation contributes to nociceptive signaling in CENP (Uniyal et al., 2022).

Rapid tolerance development to the pain-relieving effects of opioid analgesics (i.e., within 3 days of morphine treatment) is a driver for dependency and addiction. Tolerance can be defined as diminished drug efficacy/therapeutic effect following prolonged use. Our data, for the first time, indicate a slow loss of antinociceptive effects for both hot plate and tail-flick tests. Extrapolation of tolerance/no response via the regression analysis was predicted in the hot plate to occur around day 23 (predicted *x*-intercept) and day 18 in the tail-flick assay. Interestingly, we found “partial tolerance” to the anti-allodynic effects of decursinol after only 3 days of once-daily treatment in mice with cisplatin-evoked neuropathy. However, there was no evidence of complete tolerance until day 13. In contrast to decursinol-mediated antinociception/anti-allodynia, there is no evidence of tolerance development to the hypothermic effects of decursinol in non-neuropathic mice. Similarly, while we do see evidence of “partial tolerance” to decursinol-induced hypothermia after 3 days of treatment in neuropathic mice, neuropathic mice fail to develop complete tolerance to the hypothermic effects of decursinol following 14 days of once-daily treatment despite decursinol inducing an ∼15% change in body temperature. As decursinol appears to have a greater effect and faster tolerance development in mice with neuropathic pain, it is possible that the pain-relieving effects of decursinol are likely to be mediated by inhibiting neuroinflammation, a hallmark of neuropathic pain (Skaper et al., 2018). Studies found elevated levels of the pro-inflammatory cytokines TNF-α, IL-1β, and IL-6 in the periaqueductal grey (PAG) of rats following the induction of CENP with oxaliplatin (Xu et al., 2018), and that decursinol pretreatment severely attenuated pro-inflammatory cytokine responses, including licking, biting, and scratching, in mice following intrathecal administration of TNF-α, IL-1β, and IFN-γ (IL-6 was not tested; Choi et al., 2003b). Similarly, decursinol angelate (DA), an AGN-derived compound, inhibited pro-inflammatory cytokines associated with cancer cell proliferation, including TNF-α (Kim et al., 2010) while decursin blocked lipopolysaccaride-induced expression of MCP-1, IL-8, TNF-α, and IL-1β in cells and attenuated IL-1β and TNF-α (Kim et al., 2006). Taken together, these studies suggest that the effects of AGN-derived compounds may mediate their analgesic effects by inhibiting neuroinflammation induced by pro-inflammatory cytokines. Future studies should assess whether mice treated with ducursinol show an upregulation of pro-inflammatory cytokines following CENP, and if co-administration of decursinol attenuates upregulation of these cytokines concomitant with its anti-allodynic effects.

A caveat for data interpretation of the different pain models was the observed sedation and ataxic effects of decursinol at doses greater than 30 mg/kg. However, given that doses of decursinol that did not induce ataxia (3 and 10 mg/kg) did exert anti-allodynia effects in mice with neuropathic pain, its pain-killing efficacy in this model was likely less confounded than for the acute pain models. Thus, we looked at tolerance development to the ataxic effects of decursinol (50 mg/kg) in non-neuropathic mice and found that tolerance did not develop to the ataxic effects of decursinol with mice still showing profound ataxia on the rotarod despite evidence of complete antinociceptive tolerance in the tail-flick assay. Furthermore, if decursinol-induced ataxia was solely responsible for the attenuation of nociceptive behaviors, it is unlikely that decursinol or other related AGN-derived compounds would affect inflammatory processes: inhibiting levels of pro-inflammatory cytokines (TNF-α and IL-1β; Kim et al., 2006; 2010; Ma et al., 2009) while simultaneously increasing levels of anti-inflammatory cytokines (IL-4 and IL-13; Lee et al., 2020). Taken together, these data suggest that AGN compounds, including decursinol, do have analgesic effects independent of decursinol-induced ataxia.

In summary, we found that decursinol induced antinociception in acute models of thermal pain (hot plate and tail-flick) and reversed allodynia in a model of cisplatin-evoked neuropathic pain. Decursinol seemed to be more effective in a model of neuropathic pain than in acute pain models. As with other analgesics, tolerance occurred to the antinociceptive and anti-allodynic effects of decursinol, and our data indicated that the anti-allodynic effects of decursinol might be, at least in part, mediated through opioid signaling. Given that decursinol has strong oral bioavailability and seems to be especially efficacious in reversing allodynia in a neuropathic pain model, clinical trials are warranted to study its utility as a source of metabolic production of decursinol both alone and as an adjuvant with other analgesics, as a viable non-opioid alternative for managing chronic pain.

## Data Availability

The raw data supporting the conclusions of this article will be made available by the authors, without undue reservation.

## Abbreviations

%ΔBT, Percent change in body temperature; 5-HT2, Serotonin receptor 2; AGN, Angelica gigas Nakai; CB1, Cannabinoid receptor type-1; CB2, Cannabinoid receptor type-2; CENP, Chemotherapy-evoked neuropathic pain; GPCR, G protein-coupled receptor; IP, Intraperitoneal; MOR, Mu opioid receptor; %MPE, maximal possible effect; SC, Subcutaneous; WT, Wild-type.
